# Association between Gallstone Disease and Statin Use: A Nested Case—Control Study in Korea

**DOI:** 10.3390/ph16040536

**Published:** 2023-04-03

**Authors:** Mi Jung Kwon, Jung Woo Lee, Ho Suk Kang, Hyun Lim, Eun Soo Kim, Nan Young Kim, Hyo Geun Choi, Min-Jeong Kim

**Affiliations:** 1 Department of Pathology, Hallym University Sacred Heart Hospital, Hallym University College of Medicine, Anyang 14068, Republic of Korea; 2 Department of Surgery, Hallym University Sacred Heart Hospital, Hallym University College of Medicine, Anyang 14068, Republic of Korea; 3 Division of Gastroenterology, Department of Internal Medicine, Hallym University Sacred Heart Hospital, Hallym University College of Medicine, Anyang 14068, Republic of Korea; 4 Department of Radiology, Hallym University Sacred Heart Hospital, Hallym University College of Medicine, Anyang 14068, Republic of Korea; 5 Hallym Institute of Translational Genomics and Bioinformatics, Hallym University Medical Center, Anyang 14068, Republic of Korea; 6 Suseo Seoul ENT Clinic and MD Analytics, 10, Bamgogae-ro 1-gil, Gangnam-gu, Seoul 06349, Republic of Korea

**Keywords:** gallstone disease, statins, lipophilic statin, hydrophilic statin, nested case—control study

## Abstract

The correlation between statin use and the development of gallstone disease remains controversial. Existing data, primarily based on Caucasian populations, are biased, thus necessitating validation studies involving Asian cohorts. We conducted a nested case–control study using data from the Korean National Health Insurance Service Health Screening Cohort (2002–2019) to determine the likelihood of gallstone disease according to periods of previous statin use and type of statin. Among the 514,866 participants, 22,636 diagnosed with gallstones at ≥2 clinic visits (using the International Classification of Diseases, 10th revision, code K80) were matched 1:4 to 90,544 controls for age, sex, income, and residential area, and their statin prescription history for 2 years prior to the index date was examined. Propensity-score-weighted odds ratios (ORs) for gallstone disease were calculated using conditional logistic regression. Long-term use (>545 days) of any statin or lipophilic statins was associated with lower odds of incident gallstones (OR = 0.91, 95% confidence interval [CI] = 0.86–0.96, *p* < 0.001 and OR = 0.88, 95% CI = 0.83–0.93, *p* < 0.001, respectively) after adjusting for confounders. Short-term use (180–545 days) of any statin or hydrophilic statins was not statistically related to incident gallstones. In summary, prior statin medication, particularly long-term lipophilic statin use, may confer a preventive advantage against gallstone disease.

## 1. Introduction

Gallstone disease (cholelithiasis) imposes a significant epidemiological and economic burden worldwide [1,2,3]. Patients with gallstone disease are at increased risk of developing cholecystitis, cholangitis, biliary pancreatitis, and even gallbladder cancer, all of which lead to increased risk of mortality [1]. Considering the close relationship between gallstone disease and a high-saturated fat and meat diet [4], this disease is characterized by considerable variations in prevalence rates among different geographic areas and ethnicities; these range from 10.7% in Asia to 21.9% in Europe and 28.5% in North America [2,5,6,7,8,9]. Gallstone disease is a significant cause of surgery that has substantial socioeconomic costs in most Western countries, with annual spending of $6.5 billion reported in the United States (US) [3,10] and $0.51 million in 2013 in a single city in Brazil [11]. Despite a relatively low prevalence (2.0–3.43%) in the Republic of Korea (hereafter, Korea) [12,13], the pattern of gallstone disease has been analogous to that in Western countries in recent years [14]. With an approximately three-fold increase in the number of patients admitted to hospitals between 1996 and 2015 [15], gallstone disease represents a major health burden in terms of hospital admissions and critical care in Korea [12,15,16]. Taking into account Korea’s aging society and lifestyle alterations over several decades owing to its rapid development [4], modifiable risk factors for gallstone disease are of immense public health importance for preventive health initiatives.

Gallstone disease risk factors include genetics and lifestyle changes, comorbid conditions, dietary habits, and medication use [4]. Advanced age, female sex, Caucasian race, family history of gallstones, and specific metabolic risk factors including fatty liver disease, obesity-associated conditions, and insulin resistance may escalate the risk of gallstone disease [12,17,18]. Regarding medication, statins impede cholesterol gallstone formation in animal models [19,20], disintegrate gallstones in humans [21], and diminish the risk of surgical intervention due to gallstone disease, indicating their potential role in the prevention of gallstones [22]. Statins are the major prescribed lipid-lowering agents for treating hypercholesterolemia and cardiovascular disease, and act as competitive inhibitors of 3-hydroxy-3 methylglutaryl-coenzyme A (HMG-CoA) reductase, the rate-limiting enzyme that regulates the synthesis of cholesterol in the mevalonate pathway [23]. Enhanced cholesterol nucleation caused by dysregulated cholesterol metabolism is the major cause of gallstone genesis, constituting 75–80% of cases [24]. Statins play a preventive role in gallstone formation and recurrence and subsequent complications [25,26,27,28,29]. Although these drugs are perceived to be safe, several studies have reported that lipid-lowering medication enhances the risk of gallstone disease and other conditions such as statin-induced acute pancreatitis [30,31,32], and that statins may be involved in immune-mediated inflammatory reaction, direct cellular toxicity, and metabolic effects [33,34,35].

Despite increasing interest in the risk-to-benefit ratio of statins over the past decade, the effect of statins on human gallstone disease have been controversial, with only ten primary studies on this topic [25,26,27,28,29,31,36,37,38,39]. These publications were primarily focused on Western populations, with limited cohort group matching mostly based on age and sex, and limited adjustments for confounders [25,26,27,28,29,31,37,38]. These factors do not allow for the generalization of previously reported results. Moreover, these studies lack cohort data from universal national healthcare databases. One meta-analysis indicated an overall reduction of 14% in the probability of gallstone disease in statin users, on the basis of six well-designed studies with a prominent level of heterogeneity [40]. No significant results were identified in a Taiwanese-based study (1014 gallstone cases and 1014 controls matched by age, sex, and index date) nor in a single Asian population-based study [36], indicating that further validation studies are required. As statins are broadly used worldwide, it is essential to elucidate their impact on the hazard of cholelithiasis, one of the most common digestive disorders, through validation studies on Asian populations.

We hypothesized that statin use lessens the incidence of gallstone disease in the Korean population. This study expands on previous research that investigated the potential risk factors involved in statin use relating to statin type and duration of medication, using a nationwide public healthcare dataset with a precisely matched nested case–control design. Using a validated Korean healthcare database and adjusting for potential confounding factors, we explored the probability of incident gallstone disease in patients who had previously used statins compared with a matched control group.

## 2. Results

### 2.1. Baseline Characteristics of the Study Participants

The current study included 22,636 patients with gallstones and 90,544 control participants. When the patient and comparison cohorts were propensity-score matched, the groups had similar demographic features (income, region of residence, sex, and age; standardized difference = 0) (Table 1). The proportion of patients with a Charlson Comorbidity Index (CCI) score ≥2 and a history of dyslipidemia was higher in the gallstone group than in the counterpart group. The remaining features, including weight status, smoking status, alcohol consumption, systolic blood pressure (SBP), hemoglobin, fasting blood glucose, diastolic blood pressure (DBP), and total cholesterol, were also similarly balanced between the gallstone and non-gallstone counterpart groups (standardized difference ≤0.2).

### 2.2. Odds Ratios of Incident Gallstones for the Duration of Use and Type of Statin

**Figure 1:**
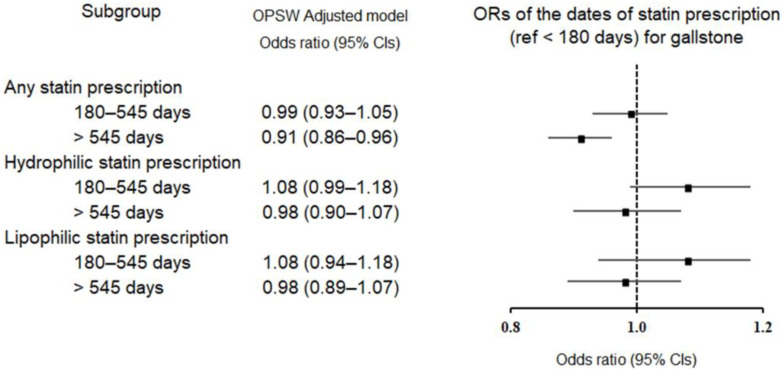
Forest plot depicting overlap propensity-score-weighted (OPSW) odds ratios (95% confidence intervals [CIs]) of incident gallstones according to statin type and duration of statin prescription. The reference period is <180 days.

The odds of subsequently occurring gallstones were investigated with respect to the duration of use of any statin and statin type (hydrophilic vs. lipophilic) (Table 2 and Figure 1). Long-term medication (>545 days) with any statin or lipophilic statins was associated with a lower likelihood of developing gallstones (odds ratio [OR] = 0.91, 95% confidence interval [CI] = 0.86–0.96, *p* < 0.001 and OR = 0.88, 95% CI = 0.83–0.93, *p* < 0.001, respectively). In contrast, short-term use (180–545 days) of any statin or hydrophilic statins exhibited no statistical relationship with incident gallstones.

## 3. Discussion

Although a growing number of studies have demonstrated the influence of statin use on the risk of gallstones in the European population [28,29,31,37], large-scale nationwide studies based on Asian patient groups are limited [36], and the effects of statin use on incident gallstones remain unclear [31,36]. In this large nationwide cohort study, we observed that previous statin prescription, especially lipophilic statin use for a long period (>545 days), was associated with reduced risk of incident gallstones after adjusting for potential confounders. Thus, this finding seems to support a potential relationship between statin administration and the decrease in incident gallstones in the adult Korean population. This is supported by the results of an earlier large study in the United Kingdom (UK) published in 2009 that comprised 27,035 patients in the gallstone cohort and 106,531 controls (OR = 0.78, 95% CI = 0.73–0.83) [28], and also by the results of a 2010 case–control study in Israel comprising 1465 patients with gallstones and 5860 controls (OR = 0.69, 95% CI = 0.57–0.84) [26]. Statin use was associated with an 18% decline in the risk of cholecystectomy for gallstone disease in a female cohort study (*n* = 2479) carried out as part of a prospective nurses’ health study in the US (95% CI = 0.70–0.96) [27]. Furthermore, in the latest clinical trial in the Netherlands involving patients who had undergone bariatric surgery, which revealed that postoperative weight loss predisposed them to gallstone disease, patients receiving statins had a 58% lower likelihood of gallstone development than those who did not receive statins (95% CI = 0.20–0.90) [29]. These findings suggest the potential disease-prevention and -inhibition implications of statin use.

However, previous population-based studies in France and Taiwan did not observe a meaningful association between incident gallstones and statin use [31,36]. Furthermore, another large cohort study based on American military healthcare data (13,626 statin users and 29,812 controls) reported that statin administration was unrelated to the risk of gallstones [39]. The differences in results can be attributed to variations in the study cohorts, study design, and/or methods of statistical analysis. To reduce the effects of confounding factors, our study used well-structured nationwide data with adjustment for possible confounding variables. Therefore, we were able to reproduce previous findings showing the preventive potential of prior statin medication against gallstones.

Notably, long-term users were less likely to develop gallstones than short-term users or non-users. In the present study, participants using any statin or lipophilic statins exhibited 9% and 12% reductions (95% CI = 0.86–0.96 and 95% CI = 0.83–0.93) in the probability of incident gallstones, respectively, only when the statins were administered for a long duration (>545 days); conversely, short-term use (180–545 days) of statins showed no statistically significant association with gallstones. Despite the different duration cut-off values, a case-control study in Switzerland showed that statin administration for >180 days was related to a 23% lower risk of gallstone disease in the treatment group than in the counterpart group (95% CI = 0.65–0.92), with no such effect observed for statin use for ≤180 days [25]. A study based on a northern Denmark population, one of the most extensive cohort studies involving gallstone (*n* = 32,494) and control (*n* = 324,925) groups, indicated that statin use for either 1–2 years or ≥3 years had a protective effect, with 11% and 24% lower likelihoods of gallstone disease, respectively, in the patient group than in the comparison group, revealing a correlation between increased duration of statin use and decreased odds for gallstones [37]. In contrast, individuals who had recently started taking statins exhibited a 1.17-fold greater risk of gallstone development (95% CI = 1.06–1.30) [37]; opposite correlations were noted in the UK case–control study (OR = 1.19, 95% CI = 1.07–1.32) [28]. Overall, statin use seems to have a cumulative protective effect on patients who have undergone bariatric surgery; the dose-effect relationship between statin use and the formation of gallstones is remarkable, depending on the surgery method, possibly because of imbalanced digestive absorption in the operated gastrointestinal tract [29,41]. The relatively low ORs in our study compared with those reported in European population-based studies might be due to the higher prevalence of cholesterol gallstones and the lower proportion of pigment gallstones in Western populations compared with Asian people [42]. Although the pattern of gallstone disease in Korea is similar to that observed in Western countries in the last 20 years [14], the ratio of cholesterol stones is slightly lower than that of pigment stones, at 0.96:1 [16], which may have contributed to the low ORs in the present study.

Cholesterol gallstones can develop due to various factors, including hypersecretion of bile, genetic factors, inflammation, mucin accumulation in the gallbladder, sluggish intestinal motility, enhanced cholesterol absorption in the gut, and changes in gut microbiota [24]. Metabolic conditions such as insulin resistance, type 2 diabetes, and obesity can also lead to inflammation and an imbalance in inflammatory cytokines [24,43], which may contribute to the development of gallstones [44]. By inhibiting HMG-CoA reductase, statins can reduce hepatic cholesterol biosynthesis and biliary cholesterol secretion, thereby preventing cholesterol gallstone disease [19,20,45]. Additionally, statins possess pleiotropic properties such as anti-inflammatory, immunomodulatory, antifungal, antimicrobial, antioxidant, and anticancer effects that can further stabilize risk factors for gallstone formation in patients with metabolic conditions [43,44,46]. Combination therapy with simvastatin and ezetimibe reduces plasma levels of remnant cholesterol, apolipoprotein B/lipoprotein, triglycerides, and cholesteryl esters in patients with gallstones, potentially due to the anti-inflammatory effects of statins [44].

The association of statins with incident gallstones may vary between the different statin types. We noted that previous use of lipophilic statins reduced the probability of gallstones, but no such link was observed with hydrophilic statin use. Scarce data are available on the preventive effects of different statin types against gallstones. The possible reasons for this are illustrated by a few experimental and clinical studies. Although an animal study failed to demonstrate a significant reduction in total cholesterol and gallstone formation in animals treated with simvastatin (lipophilic statin) [45], a preliminary clinical study suggested that lovastatin (lipophilic statin), either alone or in combination with bile acid agents (e.g., ursodeoxycholic acid), may help dissolve gallstones in humans, which may reduce biliary cholesterol levels via multiple mechanisms; lovastatin induced a 28% response rate to dissolution therapy [21]. Additionally, a clinical trial showed that adding simvastatin to ezetimibe reduced cholesterol particles in gallstones [44]. Lipophilic statins may have more beneficial outcomes than hydrophilic statins in terms of anti-inflammatory effects and immune suppression, which may be effectually related to a reduction in the risk of gallstone development [43,44,47]. However, Erichsen et al. [37] reported no significantly different ORs according to statin subtype, although the authors did not describe their results in detail. Patients with type IIa hyperlipidemia medicated with 10 mg/day pravastatin (hydrophilic statin) for 12 months showed enhanced bile pathogenicity even at a relatively low dose, and pravastatin could reduce the incidence and complications of cholesterol gallstones [43]. Nevertheless, further research on this topic is necessary.

The strengths of this study include its extensive and balanced gallstone (*n* = 22,636) and control cohorts (*n* = 90,544) extracted from a well-organized nationwide healthcare database, which helped minimize the risk of selection bias and mimic the rigor of a randomized trial. As the Korean National Health Insurance Service Health Screening Cohort (KNHIS-HSC) database contains data from each hospital and clinic in the country, no medical history was excluded during the follow-up period, which may enhance the generalizability of our findings. This study used universally applied nationwide data, while previous studies were based on comparatively small regions or limited sets of medical records [25,27,31,38]. To minimize selection bias and the effects of confounding factors, the non-gallstone counterpart group was randomly chosen using propensity-score matching. Furthermore, we included many important potential confounders. The main strength of this study is the balanced distribution of socioeconomic factors (e.g., income and residential region) as well as lifestyle factors and chronic medical conditions possibly connected to gallstone or statin use (e.g., smoking status, blood pressure, fasting blood glucose levels, total cholesterol, alcohol consumption, and weight) between the patient and control groups. Large-scale, refined observational studies that adjust for the effect of confounding factors may provide important evidence on the association between statin use and gallstone disease. To our knowledge, this is the first Korean cohort study to evaluate the association between statin use and gallstone disease using propensity-score matching. The results may support the protective effect of statin use on incident gallstones.

The limitations of the present study must be considered. First, we used days of prescription to assess duration of treatment, but actual medication intake was not monitored as patient compliance could not be assessed. Second, although we tried to include confounding factors as adjustments, some factors may have been missed. Because we could not control whether participants used hydrophilic or lipophilic statins, we adjusted for each medication as a covariate in the analysis to remove possible confounding effects. Third, we did not consider statin dose or adjustment for other cholesterol-lowering drugs such as ezetimibe. Fourth, information about family history or genetic factors, type of gallstone (cholesterol vs. pigment), co-medication, diet, type or quantity of alcohol consumed, and physical activity level were not considered in the health insurance data.

In summary, our results indicate that long-term medication with statins (>545 days), particularly lipophilic statins, may reduce the likelihood of gallstone onset in the Korean population. Using Korean insurance data, this nationwide cohort study adds supportive evidence for the protective effect of statins against gallstone disease.

## 4. Materials and Methods

### 4.1. Ethical Consideration and Data Source

All analyses adhered to the guidelines and regulations of the ethics committee of Hallym University, which approved this study (2019-10-023). The need for written informed consent was waived by the Institutional Review Board.

The present retrospective, nested case–control study was based on analysis of the KNHIS-HSC database, which offers population-based data on Koreans for research purposes, by means of a random sampling method. The KNHIS is a mandatory nationwide health insurance policy in Korea that has included registration of over 98% of all Korean citizens since 1999. The medical data files and all individuals’ information were de-identified and anonymized [48]. The KNHIS-HSC database uses the diagnostic codes of the International Classification of Diseases, 10th Revision, Clinical Modification (ICD-10-CM). A detailed description of the KNHIS-HSC data has been reported in previous studies [46,49].

### 4.2. Study Population and Participant Selection

**Figure 2:**
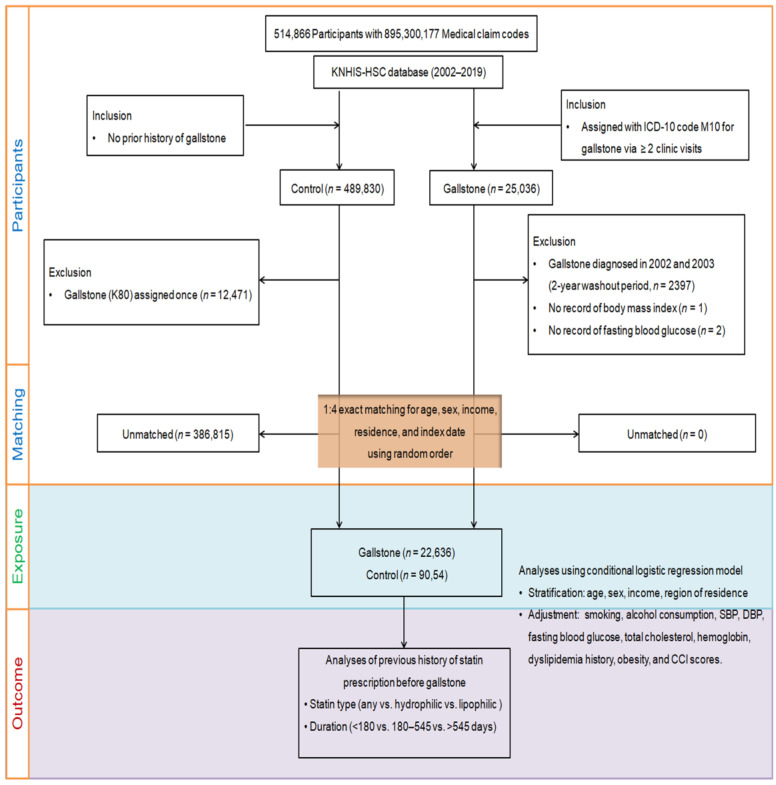
A schematic illustration of the participant selection process. Of a total of 514,866 participants, 22,636 participants with gallstones were matched to 90,544 control participants for age, sex, income, and region of residence. KNHIS-HSC, Korean National Health Insurance Service Health Screening Cohort; ICD-10, International Classification of Diseases, 10th revision; SBP, systolic blood pressure; DBP, diastolic blood pressure; CCI, Charlson Comorbidity Index.

Participants (*n* = 25,036) were selected from 514,866 adult patients (age >40 years) with 895,300,177 medical claim codes in the KNHIS-HSC database from 2002 through 2019 (Figure 2). To reduce the incidence of false positives, records of patients with gallstones diagnosed using ICD-10 code K80 following more than two clinic visits were retrieved. The index date for every patient with gallstones was established as the day when the ICD-10 code was electronically assigned to the participant in the health insurance claims dataset. To avoid including in the analysis individuals with pre-existing gallstones that occurred before the index date, we excluded patients who were diagnosed with gallstones in 2002 and 2003 (2 years of washout period, *n* = 2397). Participants with no data for body mass index (kg/m^2^, *n* = 1) or fasting blood glucose level (*n* = 2) were discounted from the gallstone group. The counterpart non-gallstone group included participants without gallstones between 2002 and 2019 (*n* = 489,830). Comparison participants were discounted if they had been diagnosed with ICD-10 code K80 (gallstone) at least once (*n* = 12,471).

To optimize the balance of baseline characteristics between the gallstone and comparison groups, propensity-score matching was applied based on residential region, income, age, and sex, and the data were sorted employing a random number order, followed by selection from top to bottom to prevent possible selection bias. As the index date of the control participants depended on the index date of their matched counterparts with gallstones, each patient with gallstones and each matched control member had the same index date. Therefore, we ruled out participants in the control group who died before the index date. During the matching step, 386,815 control members were discounted. Thus, 22,636 patients with gallstones were matched 1:4 to 90,544 comparison individuals. We then surveyed patients in both groups who had a history of statin prescription before the index date.

### 4.3. Independent Variable (Prescription Date of Statin Medication)

We retrospectively identified the statin prescription dates prior to the index date, for both groups. Patients were considered statin users if they had a statin prescription history within 2 years of the index date [27,29]. Those with any previous statin history >2 years from the index date in their medical records were ruled out. To estimate the cumulative impact of statin exposure for each individual, the total duration of drug use was computed as the total number of prescription days in the 2 years preceding the index date and was categorized into three classes: Class 1: <180 days, Class 2: 180–545 days, and Class 3: >545 days. Statin users were defined as patients who had statin prescriptions for a minimum of 180 days [50,51]; those with prescriptions for <180 days, 180–545 days, and >545 days were deemed statin non-users, short-term users, and long-term users, respectively, as previously explained [29,46].

Simvastatin, atorvastatin, lovastatin, pravastatin, fluvastatin, and rosuvastatin were the prescribed statins in this study, and were grouped as lipophilic (fluvastatin, lovastatin, simvastatin, and atorvastatin) or hydrophilic statins (rosuvastatin and pravastatin) in order to evaluate the impact of statin type on the occurrence of gallstones according to the statins’ lipid affinity.

### 4.4. Dependent Variable (Gallstone Formation)

Patients with more than two clinic visits for treatment of gallstones defined according to ICD-10 code K80 (cholelithiasis) were deemed as having gallstones.

### 4.5. Covariates

Age was divided into 10 subgroups with 5-year intervals, from 40–44 years to ≥85 years. The five income groups ranged from Class 1 (lowest income) to 5 (highest income). The region of residence was divided into urban (Seoul, Incheon, Daejeon, Daegu, Gwangju, Ulsan, and Busan) and rural areas (Chungcheongbuk, Chungcheongnam, Gyeonggi, Gangwon, Gyeongsangbuk, Gyeongsangnam, Jeollabuk, Jeollanam, and Jeju).

Tobacco smoking was categorized according to the participant’s current smoking status (non-smoker, past smoker, or current smoker). Alcohol consumption was categorized based on frequency (<1 time/week or ≥1 time/week). Obesity was defined using body mass index (kg/m^2^), which was categorized as <18.5 (underweight), ≥18.5 to <23 (normal), ≥23 to <25 (overweight), ≥25 to <30 (obese I), or ≥30 kg/m^2^ (obese II) according to the Asia-Pacific criteria of the Western Pacific Regional Office 2000. Data were retrieved for fasting blood glucose (mg/dL), SBP (mmHg), total cholesterol (mg/dL), DBP (mmHg), and hemoglobin (g/dL).

The CCI is commonly utilized to calculate disease burden by evaluating the presence and severity of 17 comorbidities. The CCI for each participant was summed as the total score of continuous variables from 0 (no comorbidities) to 29 (multiple comorbidities) in order to quantify the severity and number of diseases among the 17 comorbidities [52]. For patients with gallstones, history of dyslipidemia (ICD-10 code: E78) was added to the CCI if they had been treated ≥2 times.

### 4.6. Statistical Analyses

Categorical data were summarized as percentages, and standardized differences were exploited to compare the general characteristics between the cohorts. Propensity-score matching was conducted to facilitate exact matching between the gallstone and control groups for age, sex, income status, and region of residence [53]. During propensity-score matching, a greedy, nearest-neighbor matching algorithm was applied to form dyads of gallstone cases and counterparts whose propensity scores were closest to the patients’ [54]. To reduce the possibility of intergroup bias, we assessed whether the matched data were balanced in terms of absolute standardized differences in the covariates. A standardized difference of ≤0.20 in the ascertained covariate indicated an appropriate balance.

To analyze the OR with 95% CI for gallstones with statin prescriptions, conditional logistic regression was applied in the matched groups. For the logistic regression analysis, model 1 (SBP, DBP, total cholesterol, fasting blood glucose, hemoglobin, and dyslipidemia history) and model 2 (model 1 plus obesity, smoking status, alcohol consumption, statin type, and CCI scores) were considered (when a lipophilic statin was analyzed, hydrophilic statin was adjusted as a covariate, and vice versa). Additionally, the effect of statin prescription was analyzed by division into hydrophilic and lipophilic statins. Two-tailed analyses were performed, and statistical significance was defined as *p* < 0.05. SAS version 9.4 (SAS Institute Inc., Cary, NC, USA) was used for all statistical analyses.

## 5. Conclusions

In summary, our results may indicate that long-term use of statins (>545 days), particularly lipophilic statins, could have a beneficial effect against the onset of gallstone disease in the Korean population. Based on Korean insurance data, this nationwide cohort study adds supportive evidence for the protective effect of statin use against gallstone disease.

## Figures and Tables

**Table 1:** General characteristics of participants.

Characteristics	Total Participants		
	Gallstone (*n*, %)	Control (*n*, %)	Standardized Difference
Age (years)			0.00
40–44	197 (0.87)	788 (0.87)	
45–49	1199 (5.30)	4796 (5.30)	
50–54	2454 (10.84)	9816 (10.84)	
55–59	3983 (17.60)	15,932 (17.60)	
60–64	3890 (17.19)	15,560 (17.19)	
65–69	3560 (15.73)	14,240 (15.73)	
70–74	3043 (13.44)	12,172 (13.44)	
75–79	2388 (10.55)	9552 (10.55)	
80–84	1390 (6.14)	5560 (6.14)	
85+	532 (2.35)	2128 (2.35)	
Sex			0.00
Male	12,845 (56.75)	51,380 (56.75)	
Female	9791 (43.25)	39,164 (43.25)	
Income			0.00
1 (lowest)	3760 (16.61)	15,040 (16.61)	
2	2697 (11.91)	10,788 (11.91)	
3	3363 (14.86)	13,452 (14.86)	
4	4710 (20.81)	18,840 (20.81)	
5 (highest)	8106 (35.81)	32,424 (35.81)	
Region of residence			0.00
Urban	9583 (42.34)	38,332 (42.34)	
Rural	13,053 (57.66)	52,212 (57.66)	
Obesity †			0.15
Underweight	476 (2.10)	2509 (2.77)	
Normal	6771 (29.91)	31,932 (35.27)	
Overweight	6063 (26.78)	24,724 (27.31)	
Obese I	8360 (36.93)	28,692 (31.69)	
Obese II	966 (4.27)	2687 (2.97)	
Smoking status			0.04
Nonsmoker	14,897 (65.81)	60,165 (66.45)	
Past smoker	4076 (18.01)	15,127 (16.71)	
Current smoker	3663 (16.18)	15,252 (16.84)	
Alcohol consumption			0.02
<1 time a week	14,039 (62.02)	55,202 (60.97)	
≥1 time a week	8597 (37.98)	35,342 (39.03)	
Systolic blood pressure			0.00
<120 mmHg	6291 (27.79)	25,549 (28.22)	
120–139 mmHg	11,334 (50.07)	44,900 (49.59)	
≥140 mmHg	5011 (22.14)	20,095 (22.19)	
Diastolic blood pressure			0.01
<80 mmHg	10,838 (47.88)	43,411 (47.94)	
80–89 mmHg	8150 (36.00)	32,096 (35.45)	
≥90 mmHg	3648 (16.12)	15,037 (16.61)	
Fasting blood glucose			0.08
<100 mg/dL	12,401 (54.78)	53,317 (58.89)	
100–125 mg/dL	7327 (32.37)	27,881 (30.79)	
≥126 mg/dL	2908 (12.85)	9346 (10.32)	
Total cholesterol			0.08
<200 mg/dL	13,237 (58.48)	50,095 (55.33)	
200–239 mg/dL	6722 (29.70)	28,919 (31.94)	
≥240 mg/dL	2677 (11.83)	11,530 (12.73)	
Hemoglobin			0.02
≥12 g/dL for men and ≥10 g/dL for women	21,963 (97.03)	88,448 (97.69)	
<12 g/dL for men and <10 g/dL for women	673 (2.97)	2096 (2.31)	
CCI score			0.23
0	9676 (42.75)	53,553 (59.15)	
1	4678 (20.67)	15,001 (16.57)	
≥2	8282 (36.59)	21,990 (24.29)	
Dyslipidemia history	13,832 (61.11)	48,958 (54.07)	0.22
Duration of statin prescription			0.13
<180 days	17,996 (79.50)	74,165 (81.91)	
180–545 days	1811 (8.00)	6395 (7.06)	
>545 days	2829 (12.50)	9984 (11.03)	
Duration of hydrophilic statin prescription			0.03
<180 days	21,267 (93.95)	85,997 (94.98)	
180–545 days	685 (3.03)	2238 (2.47)	
>545 days	684 (3.02)	2309 (2.55)	
Duration of lipophilic statin prescription			0.12
<180 days	19,157 (84.63)	78,122 (86.28)	
180–545 days	1587 (7.01)	5486 (6.06)	
>545 days	1892 (8.36)	6936 (7.66)	

Abbreviations: CCI, Charlson Comorbidity Index. † Obesity (body mass index, kg/m^2^) was categorized as <18.5 (underweight), ≥18.5 to <23 (normal), ≥23 to <25 (overweight), ≥25 to <30 (obese I), and ≥30 (obese II).

**Table 2:** Crude and overlap propensity-score-weighted odds ratios of incident gallstones according to statin type and duration of statin prescription.

Characteristics	Gallstone	Control	Odds Ratios of Incident Gallstones (95% Confidence Interval)			
	(*n* Exposure/ Total, %)	(*n* Exposure/ Total, %)	Model 1 † ‡	*p*-Value	Model 2 † §	*p*-Value
Any statin prescription						
<180 days	17,996/22,636 (79.50)	74,165/90,544 (81.91)	1		1	
180–545 days	1811/22,636 (8.00)	6395/90,544 (7.06)	1.02 (0.97–1.08)	0.470	0.99 (0.93–1.05)	0.645
>545 days	2829/22,636 (12.50)	9984/90,544 (11.03)	0.94 (0.89–0.98)	0.007 *	0.91 (0.86–0.96)	<0.001 *
Hydrophilic statin prescription						
<180 days	19,157/22,636 (84.63)	78,122/90,544 (86.28)	1		1	
180–545 days	1587/22,636 (7.01)	5486/90,544 (6.06)	1.08 (0.99–1.18)	0.072	1.08 (0.99–1.18)	0.102
>545 days	1892/22,636 (8.36)	6936/90,544 (7.66)	0.99 (0.90–1.08)	0.737	0.98 (0.90–1.07)	0.598
Lipophilic statin prescription						
<180 days	21,267/22,636 (93.95)	85,997/90,544 (94.98)	1		1	
180–545 days	685/22,636 (3.03)	2238/90,544 (2.47)	1.04 (0.98–1.10)	0.264	1.00 (0.94–1.06)	0.976
>545 days	684/22,636 (3.02)	2309/90,544 (2.55)	0.90 (0.85–0.96)	0.001 *	0.88 (0.83–0.93)	<0.001 *

Abbreviations: CCI, Charlson Comorbidity Index; SBP, systolic blood pressure; DBP, diastolic blood pressure. * Conditional logistic regression analysis; significance at *p* < 0.05; † Stratified model for age, sex, income, and region of residence; ‡ Model 1 was adjusted for SBP, DBP, fasting blood glucose, total cholesterol, hemoglobin, and dyslipidemia history; § Model 2 was adjusted for the variables in model 1 plus obesity, smoking status, alcohol consumption, statin type, and CCI scores (when a lipophilic statin was analyzed, hydrophilic statin was adjusted as a covariate, and vice versa).

## Data Availability

All data were obtained from the database of the National Health Insurance Sharing Service (NHISS) and are available at https://nhiss.nhis.or.kr/ (accessed on 1 November 2022). The NHISS allows access to all data (downloaded from the website) for any researcher who agrees to follow the research ethics and pays a processing fee.
